# A generalized physiologically-based toxicokinetic modeling system for chemical mixtures containing metals

**DOI:** 10.1186/1742-4682-7-17

**Published:** 2010-06-02

**Authors:** Alan F Sasso, Sastry S Isukapalli, Panos G Georgopoulos

**Affiliations:** 1 Environmental and Occupational Health Sciences Institute, A joint institute of UMDNJ - Robert Wood Johnson Medical School and Rutgers University, Piscataway, New Jersey, USA; 2 UMDNJ-Robert Wood Johnson Medical School Department of Environmental and Occupational Medicine, Piscataway, New Jersey, USA; 3 Rutgers University Department of Chemical and Biochemical Engineering, Piscataway, New Jersey, USA

## Abstract

**Background:**

Humans are routinely and concurrently exposed to multiple toxic chemicals, including various metals and organics, often at levels that can cause adverse and potentially synergistic effects. However, toxicokinetic modeling studies of exposures to these chemicals are typically performed on a single chemical basis. Furthermore, the attributes of available models for individual chemicals are commonly estimated specifically for the compound studied. As a result, the available models usually have parameters and even structures that are not consistent or compatible across the range of chemicals of concern. This fact precludes the systematic consideration of synergistic effects, and may also lead to inconsistencies in calculations of co-occurring exposures and corresponding risks. There is a need, therefore, for a consistent modeling framework that would allow the systematic study of cumulative risks from complex mixtures of contaminants.

**Methods:**

A Generalized Toxicokinetic Modeling system for Mixtures (GTMM) was developed and evaluated with case studies. The GTMM is physiologically-based and uses a consistent, chemical-independent physiological description for integrating widely varying toxicokinetic models. It is modular and can be directly "mapped" to individual toxicokinetic models, while maintaining physiological consistency across different chemicals. Interaction effects of complex mixtures can be directly incorporated into the GTMM.

**Conclusions:**

The application of GTMM to different individual metals and metal compounds showed that it explains available observational data as well as replicates the results from models that have been optimized for individual chemicals. The GTMM also made it feasible to model toxicokinetics of complex, interacting mixtures of multiple metals and nonmetals in humans, based on available literature information. The GTMM provides a central component in the development of a "source-to-dose-to-effect" framework for modeling population health risks from environmental contaminants. As new data become available on interactions of multiple chemicals, the GTMM can be iteratively parameterized to improve mechanistic understanding of human health risks from exposures to complex mixtures of chemicals.

## Background

Physiologically based toxicokinetic (PBTK) models are an important class of dosimetry models that are useful in estimating internal and target tissue doses of xenobiotics for risk assessment applications [1]. PBTK models employ mass balances on compartments within a human or animal body, for the purpose of estimating the time-course profiles of toxicant concentrations in tissues and fluids. These models are also useful for understanding therapeutic outcomes from internal tissue exposures to pharmaceuticals [2]. In conjunction with epidemiological and demographic data, and models of environmental pollution and exposure, PBTK models are applied to assess population health risks and provide a scientific basis for regulating the production and use of chemicals [3]. PBTK models provide a critical mechanistic linkage between exposure models and biologically-based dose-response models. Thus, PBTK models for complex mixtures should form a central component of any human exposure and health risk modeling framework that aims to address multiple contaminants [4].

Humans are typically exposed to multiple xenobiotic chemicals, such as pharmaceuticals, cosmetics, alcohols, metals, solvents, pesticides, volatile and semi-volatile organic compounds, etc., simultaneously. For this reason, there have been efforts to incorporate metabolic interactions in PBTK models for mixtures of selected chemicals [5]. Concurrently, there have been increasing numbers of applications involving "whole-body" physiologically-based toxicokinetic (WBPBTK) models that aim to reduce model uncertainties and better characterize inter-individual variabilities [6]. These whole-body models account for all major tissues and exposure pathways, and are capable of incorporating detailed physiological data. However, comprehensive mixture modeling efforts have not been pursued in the field of toxic metal compounds, and there are currently no available PBTK models for mixtures of metals. Indeed, toxicokinetic models have only focused on individual metals separately, despite evidence of interactions of toxic metals with other toxic metals [7], with essential metals [8], and even with nonmetal pollutants [9]. Recent developments in the field of molecular biomarkers have identified toxic interactions among metals such as arsenic, lead, and cadmium (including some toxic effects that are not seen in relation to single component exposures) [7]. Though, in the long term, there is a need for developing mechanistic toxicodynamic models for mixtures of metal compounds, in the short term there is a need for a PBTK modeling system that is capable of simulating multiple interacting metals and nonmetals simultaneously. Such a system should also incorporate realistic whole-body physiology of members of both the general and of susceptible populations.

### Toxicological interactions among metals

Due to their similarities to essential metals, toxic metals are transported and eliminated through many common cellular mechanisms by "molecular mimicry" [10]. As a result, there exist toxicokinetic and toxicodynamic interactions among toxic and essential metals [7,8]. Metal absorption, elimination, and toxicokinetics should therefore be considered highly correlated for exposed individuals, with susceptibilities resulting in differential effects of multiple metals. Population susceptibilities resulting from essential element status are often a significant source of uncertainty and variability for metals risk assessment [11]. For example, iron inhibits lead and cadmium intestinal uptake due to shared absorption mechanisms [12]; conversely, toxic metals may inhibit essential element absorption [13]. Cadmium and zinc are also known to have a variety of interactions due to the metal-binding protein metallothionein [14]. Selenium may potentially alter both arsenic and methylmercury toxicity [15]. Other nutrients such as antioxidants, Vitamins A/C/E, magnesium, phosphorus, riboflavin, and methionine are also known to impact toxic metal susceptibility [16].

Low essential element status or illnesses may result in higher absorption of multiple metals [17]. This has direct implications for PBTK applications to population risk assessment, since failing to account for high correlations in the absorption of individual metals may lead to misinterpretations of biomarker data. In cases where susceptible individuals are exposed to mixtures of toxic metals while exhibiting high absorption, there is a greater likelihood of toxic effects, either due to additive or synergistic interactions. This is particularly important since some metals exhibit common toxic effects such as hepatic, renal, and neurological toxicity. Molecular biomarkers of toxic metal health effects are becoming sensitive enough to detect some toxic interactions [7]. Synergistic toxic interactions in the liver and kidneys between arsenic and cadmium [18], and lead and cadmium [19] have been observed in exposed human populations.

### Toxicological interactions among metals and nonmetals

Toxic metals affect the toxicokinetics of additional classes of chemicals such as pesticides, polychlorinated biphenyls (PCBs), polycyclic aromatic hydrocarbons (PAHs), and volatile organic compounds (VOCs). Indeed, these toxic metals can accumulate in the liver and kidneys and, due to their long half-lives, affect the hepatic and renal levels of Cytochrome P450 (CYP450) enzymes, which metabolize other xenobiotics [9]. Therefore, there is a need for a framework that links metal toxicokinetics, CYP450 dose-responses, and the subsequent impact of metals on the toxicokinetics of nonmetals. Since many PCBs, pesticides, and organic pollutants also induce or inhibit CYP450 enzymes, additional metabolic interactions are expected to occur. Table 1 lists some of the CYP450 enzymes that are affected by toxic metals, along with the classes of substrates metabolized by those enzymes. Many other effects are possible in addition to CYP450-related interactions: for example, a recent PBTK modeling study found that co-exposure to PCBs leads to an increased lactational transfer of methylmercury in mice [20].

**Table 1:** Selected interactions between metals and CYP450 enzymes in humans and animals

Metals	CYP450 effects	**Potential substrates**^**†**^	Reference
Cadmium	Induced 2A6	Carbamates, drugs	[32]
	Induced 2E1	Halogenated aliphates, triazines, organophosphates, VOCs, drugs	[32]
	Induced 2C9	Drugs, organophosphates, triazines	[32]

Lead	Inhibited 2A6	Drugs	[63]
	Inhibited 1A2 (rats)	Arylamines, organophosphates, triazines, VOCs, PCBs, drugs	[64]

Arsenic	Induced 1A1 (rats)	PAHs, VOCs, PCBs, triazines	[65,66]

Metal mixtures	Altered 1A1/2 induction by PAHs/TCDD (rats)	PAHs, VOCs, PCBs, triazines, organophosphates, drugs	[67,68]

^†^Substrate/P450 relationships from [24,69-71].

## Methods

**Figure 1:**
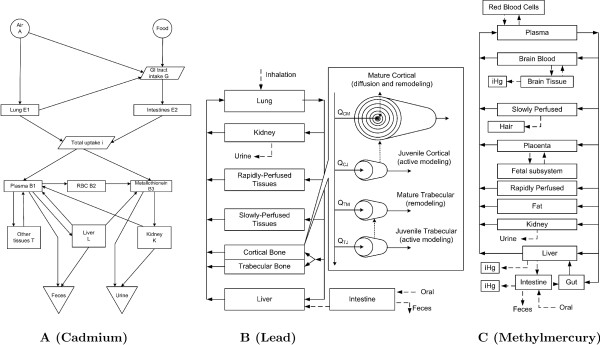
**A schematic depiction of PBTK model structures for two common toxic metals (cadmium **[33]**and lead **[45]), **and a toxic metal compound (methylmercury **[56]), **as they have been implemented in the literature**. The different physicochemical properties of the toxicants of concern have resulted in different structures (i.e. representations of the physiology) in the three models, thus limiting the usefulness of these formulations in assessing cumulative and/or comparative exposures and risks.

Despite the critical need for a multi-chemical PBTK model that considers toxic metals, discussed in the previous section, unique modeling challenges have so far prevented the implementation of such a system. The half-lives of key toxic metals in humans are highly variable, spanning time scales of days (e.g. arsenic), months (e.g. methylmercury), and decades (e.g. lead and cadmium). As shown in Figure 1, available model formulations for each metal differ greatly with respect to their basic conceptual and mathematical structures, making considerations of interaction and integration of multiple models for assessing cumulative exposures difficult or impossible. Current PBTK software platforms are not flexible enough to simultaneously allow the direct incorporation of a complex diffusion model of lead in bone, the model of pregnancy for fetal methylmercury exposure, and a biokinetic model of cadmium. However, in spite of these modeling differences, many similarities exist in the toxicokinetics of metals. The Divalent Metal Transporter 1 (DMT1) is a common gastrointestinal absorption pathway [12], and metallothionein plays an important role in overall absorption, distribution, elimination and toxicity [21]. Metabolism of metal and metalloid compounds is limited to redox reactions, methylation/demethylation, and protein conjugation [22]. Elimination of absorbed dose occurs primarily by renal excretion [23]. Such commonalities narrow the focus of the potential mixture effects to those which may have the highest impact on toxicokinetics.

### General model structure

**Figure 2:**
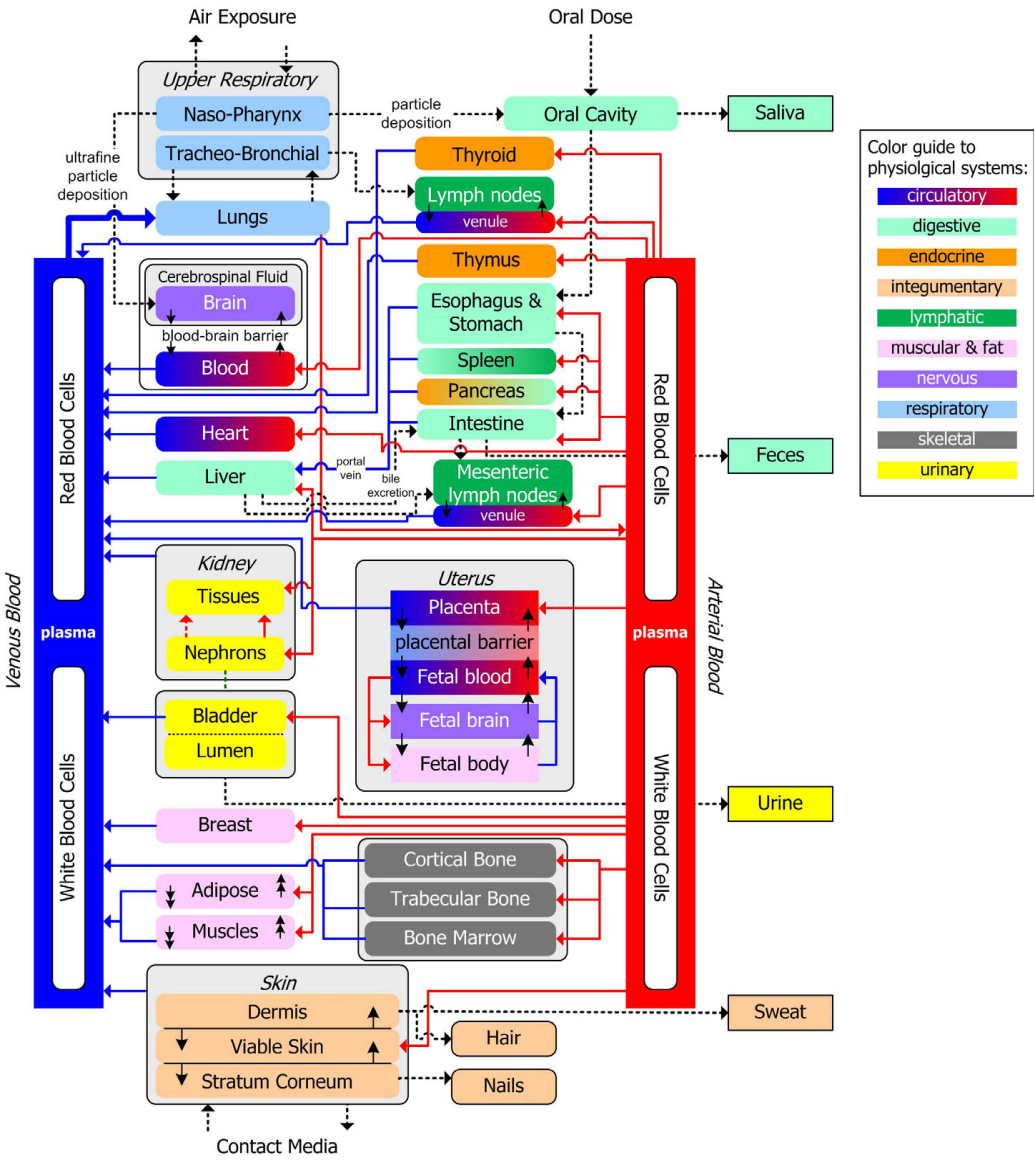
**A schematic depiction of major compartments considered in the generalized PBTK modeling framework (adapted from Georgopoulos, 2008) **[4].

Most PBTK model structures can be considered subsets of the same general "compartmentalized" or "network" physiology shown in Figure 2 (adapted from Georgopoulos, 2008 [4]). Blood flow rates and volumes of physiological compartments are (or at least should be) chemical-independent. Parameters of lumped compartments (e.g. flow rates and volumes of slowly perfused and rapidly perfused tissues) may vary based on the particular model structure and toxic endpoints of interest, and these appear as chemical-dependent. However, even these parameters need to be constrained so as to be consistent with the sum of those quantities for the remaining compartments. The model that is presented here accounts for all major tissues, and absorption and excretion mechanisms. Tissues that are not explicitly modeled in chemical-specific PBTK models can be lumped into rapidly or slowly perfused groups while maintaining overall physiological consistency. Deriving lumped parameter PBTK models from the general framework of Figure 2 reduces an artificial source of intermodel variation, maintains the structure of the original models, and does not require estimation of additional parameters. Chemical-specific PBTK models for toxic metals and nonmetals were mapped to this general formulation in the GTMM, thus allowing for simultaneous toxicokinetic modeling with metabolic interactions.

### Mathematical formulation

The general mathematical mass balance for the set of physiological compartments within the PBTK model is given by the matrix differential equation:(1)

Matrices indexed by both tissue and chemical are defined as follows: ***A ***is the matrix of chemical amounts in the different tissues; ***Q ***is the matrix of tissue flow rates; ***C***^in ^is the matrix of inlet concentrations to the tissues (typically the concentrations in the arterial blood streams, but may also be a volume-weighted average of multiple inlet streams); ***C***^out ^is the matrix of outlet concentrations; ***R ***is the matrix of net rates of metabolism for all the chemicals considered (negative values indicate formation of chemical); and ***T ***is the matrix of net rates of transport of all chemicals considered via additional processes (i.e. excretion, absorption, or inter-compartmental transfer). While the blood flows are assumed to be independent of the chemical under consideration, a chemical-specific formulation allows for selective lumping of the compartments for some chemicals.

At the tissue-level, there are several possible mass balance schemes. Chemicals may diffuse through one or more barriers and accumulate in multiple tissue regions. If a tissue is divided into extracellular and cellular subcompartments, the mass balances for chemical *i *in compartment *j *can expressed by:(2)

In the above equation, superscripts E and C denote extracellular and cellular space, respectively. *P*_*i,j *_is the tissue:blood partition coefficient, *H*_*i,j *_is the lumped permeability-area coefficient (volume/time), and  is the permeation rate of chemical through the diffusive layer (mass/time). The outlet concentration is equal to the extracellular concentration . PBTK models sometimes differ in how the driving force for diffusion is defined. If more complex transport mechanisms other than diffusion occur (i.e. carrier-mediated transport), alternative expressions for  are required.

If a chemical reaches rapid equilibrium in the tissue subcompartments, a simplified perfusion-limited assumption may be used to describe the system [24]:(3)

For the perfusion-limited assumption, the outlet concentration is equal to *C*_*i,j*_/*P*_*i,j*_. Depending on the physicochemical properties of the contaminant, PBTK models may consist entirely of diffusion- or perfusion-limited compartments, or a combination of both.

### Equations for metabolism

If metabolism is modeled as a first-order reaction, and the metabolite is an additional chemical in the PBTK model, a simple matrix multiplication solution can be used to calculate the metabolic rates of all chemicals [25]. Within each tissue, a vector of first-order metabolic rates for all chemicals is produced by the matrix multiplication **Γ **× ***y***, where Γ is the matrix of net rate constants (defined below), and ***y ***is a column vector of chemical concentrations. Here, a metabolic rate constant Γ_*B,A*_, is defined for the reaction *A *→ *B*, where rate of metabolism of *A *due to this particular pathway is Γ_*B,A*_, × *y*_A_. It follows that the formation rate of *B *is simply the negative of that for *A*. Such a representation is convenient for matrix-based computational environments. The corresponding matrix of net first-order rate constants for *N *chemical species may be defined by:(4)

For simplicity, notation for tissue index *j *has been omitted. For the case of Michaelis-Menten kinetics for a mixture of chemicals which may compete for finite enzyme sites (competitive inhibition), the kinetics may be described by [5]:(5)

where *i *and *k *denote the metabolizing and inhibiting chemical species, respectively; *V*_max,*i *_is the maximum reaction velocity (mass/time); *K*_m,*i *_is the Michaelis constant (mass/volume); *I*_*k*,*i *_is the competitive inhibition constant for chemical *k *inhibiting the metabolism of chemical *i *(mass/volume). Similar generalized equations are applicable to describe reductions in *V*_max _due to noncompetitive inhibition, or increases in *V*_max _or Γ due to enzyme induction.

### Computational implementation

The modeling system that is presented here, GTTM (Generalized Toxicokinetic Modeling System for Mixtures) was implemented in the Matlab programming environment, that has previously been reviewed as a useful tool for PBPK applications [26], and includes various toolboxes for parameter identification and visualization. Multiple diverse PBTK models may be incorporated into a common workspace, allowing for simultaneous, interacting simulations. In order to accommodate multiple chemicals and a large number of potential interactions, the GTMM utilizes matrix-based formulations. For example, every tissue is assigned a first-order reaction network matrix as shown in Equation 4, and analogous matrices address other types of reaction and transport rates. The mass balances of multiple chemicals in all the tissues are represented by a matrix of ordinary differential equations (ODEs), that are solved by the ode15s stiff ODE solver of Matlab. The inputs to the GTMM are exposure profiles, and physiological and biochemical parameters. The outputs are the time-concentration profiles of different chemicals in the various tissues. Physiological variability in the population may be consistently considered across the models for all chemicals by linking with biological databases that provide physiological values for a majority of the tissue groups. GTTM offers the option to obtain parameters from databases for the general population (i.e. the P3M physiological database [27]) and for susceptible populations (i.e. the elderly and health-impaired [28]). Other sources of whole-body physiology include the PK-Pop scaling algorithm used by PK-Sim [29], and the polynomial relationships used by PostNatal [30]. The Matlab environment allows the GTMM to generate "virtual individuals" with consistent physiology using any of the above databases.

## Results

The GTMM was evaluated with respect to its ability to predict toxicokinetics of multiple toxic metals "individually" (i.e. "one metal at a time"). Predictions of biomarkers by the GTMM were compared with the estimates from the corresponding single-metal PBTK models, using the same input data as the original literature evaluation studies of these models. For the case studies involving individual metals, the major physiological parameters for the GTMM were set to the values used in these original modeling case studies, so as to ensure direct comparison. Evaluations were performed for four toxic metals (cadmium, arsenic, lead, chromium), and a toxic metal compound (methylmercury). In all cases, the GTMM explained the available data and replicated the predictions of the various metal-specific formulations. Subsequently, the GTMM was applied to a hypothetical case involving interactions between metals and nonmetals.

### Cadmium

The general population is exposed to cadmium primarily through dietary ingestion and inhalation of cigarette smoke [31]. Kidney damage is the primary health concern; other effects include alteration of enzyme levels, liver toxicity, cancer, and hypertension [31,32]. Due to the long half-life of cadmium in humans, the PBTK formulation is different from typical PBTK formulations, as shown in Figure 1. The GTMM replicates the cadmium toxicokinetics described by the formulation by Kjellström and Nordberg (see Additional files 1 and 2) [33]. Absorbed cadmium accumulates in the kidney and liver, and binds to metallothionein proteins. Elimination from the body occurs primarily through urinary excretion, which is a slow process in humans.

**Figure 3:**
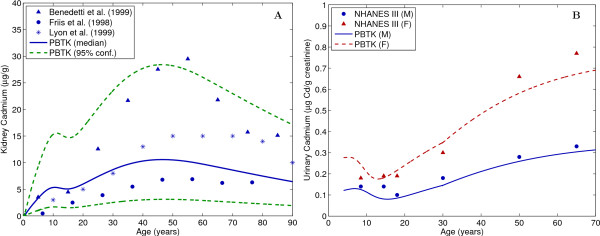
**Comparisons of GTMM predictions with measured human data from (A) autopsy measurements of kidney cadmium levels **[36-38]**and (B) urinary cadmium measurements from the National Health and Nutrition Examination Survey (NHANES) **[39]. Estimates for population exposure were obtained from Choudhury et al. (2001) [34]. All data points represent median values.

The GTMM was evaluated by applying estimates from the cadmium intake model by Choudhury et al. (2001) [34,35], and comparing to available population data. Figure 3 (A) shows comparisons to autopsy data [36-38]. Predictions were made using the median and 95th percentiles for dietary cadmium intake [34]. Data from Friis et al. (1998) [36] consist of 58 nonsmokers, while data from Lyon et al. (1999) [37] and Benedetti et al. (1999) [38] each consist of approximately 300 smokers and nonsmokers. The Benedetti data are for cadmium concentration in the whole kidney, while all other data and model predictions are for concentration in the kidney cortex. Figure 3 (B) compares model predictions to urinary data from over 12,000 individuals of the National Health and Nutrition Examination Survey (NHANES) [39]. Predictions were made assuming constant cadmium intake of 0.4 μg/kg/day, and differences between males and females are attributed to higher fractional cadmium absorption in females.

### Arsenic

**Figure 4:**
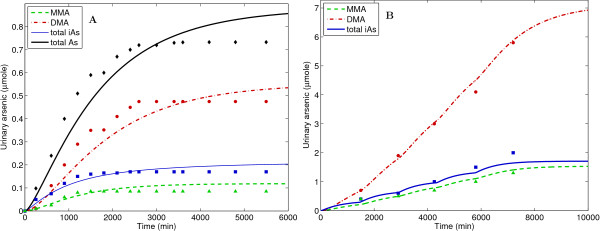
**Comparisons of GTMM predictions with measured data of cumulative urinary arsenic from a volunteer human study in which individual males ingested (A) a single 100 μg As^V ^oral dose (Lee, 1999 **[42]), **and (B) multiple 250 μg As ^III ^oral doses (Buchet et al., 1981 **[43]). Data legend: Total arsenic (black diamond), total inorganic arsenic (blue square), total MMA (green triangle), total DMA (red circle)

Arsenic is a known human carcinogen (bladder, lung, and skin), and is also linked to a variety of other toxic health endpoints. Inorganic arsenate (As^V^) and arsenite (As^III^) exist in soil and drinking water, originating from both natural and man-made sources. Organic species such as monomethylarsenic acid MMA^V ^and dimethylarsenic acid DMA^V ^exist in the environment, and are also products of inorganic arsenic metabolism in humans. While there are still uncertainties in the metabolic pathways and toxic mechanisms of each arsenical [40], the El-Masri/Kenyon PBTK model is currently the most comprehensive description of arsenic toxicokinetics in humans (see Additional files 3 and 4) [41]. Major steps in the metabolism of arsenic are (1) reduction of As^V ^to As^III^; (2) methylation of As^III ^to MMA^V^; (3) methylation of As^III ^to DMA^V^; (4) reduction of MMA^V ^to MMA^III^; (5) methylation of MMA^III ^to DMA^V^; and (6) reduction of DMA^V ^to DMA^III^. Oxidation occurs to a small extent for all species, however demethylation does not occur. Noncompetitive inhibition occurs for the methylation steps 2 and 5, since these reactions are catalyzed by arsenic (+3) methyltransferase (AS3MT). In this model, step 2 is inhibited by MMA^III ^concentration in the liver, while step 5 is inhibited by As^III^. Urinary excretion of organic and inorganic arsenic is currently the only mechanism for elimination in the model. The GTMM was evaluated against human data for single oral doses (Lee, 1999 [42]) and for repeated oral doses (Buchet et al., 1981 [43]) of inorganic arsenic. As shown in Figure 4, the GTMM was able to explain these short timescale data when applying the assumptions used for the evaluation of the arsenic-specific model [41].

### Lead

**Figure 5:**
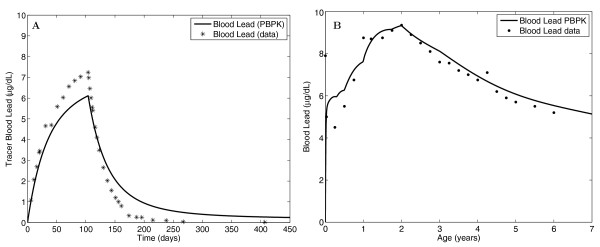
**Comparisons of GTMM predictions with measured human data of (A) tracer blood lead for a male absorbing 17.5 μg/day lead-204 for 104 days (Rabinowitz et al., 1976 **[46]), **and (B) blood lead for a subgroup of children from the Cincinnati Prospective Lead Study (Bornschein et al., 1985 **[49]), **using the O'Flaherty lead exposure model to characterize ingestion and inhalation intakes **[48]. The Cincinnati data represent the median blood lead measurements of individuals monitored from birth to early childhood, and only include children whose highest blood lead concentration did not exceed 15 μg/dL.

The general population is exposed to lead from ingestion of contaminated food and water, and from inhalation of cigarette smoke. Children are a particularly vulnerable subpopulation, as they may receive high non-dietary exposure and are more susceptible to neurotoxic effects [44]. Lead is cleared from plasma primarily by excretion into urine and uptake into bone. Approximately 95% of the lead body burden in humans is in bone, which serves as a long term reservoir for replenishment of blood lead in humans [44]. The PBTK model formulation by O'Flaherty [45] accounts for lead diffusion into several bone compartments to describe long timescales of lead bone kinetics (Figure 1). Mature cortical bone is a special case in which diffusion of lead is modeled as occurring across eight cylindrical shells in the radial direction. Short timescale performance of the GTMM was evaluated using data from a volunteer tracer lead exposure study (Rabinowitz et al., 1976 [46]), and by incorporating assumptions used by the adult lead model of O'Flaherty (1993) (See Additional file 5) [47]. Long timescale performance of the GTMM was evaluated by linking it with the O'Flaherty childhood model for lead exposure [48], and comparing results with data for a subgroup of the Cincinnati Prospective Lead Study (Bornschein et al., 1985 [49]). The model exposure parameters and corresponding data were for the subgroup of children whose blood lead concentration did not exceed 15 μg/dL [48]. As shown in Figure 5, the GTMM was able to explain both the short and long timescale data.

### Chromium

**Figure 6:**
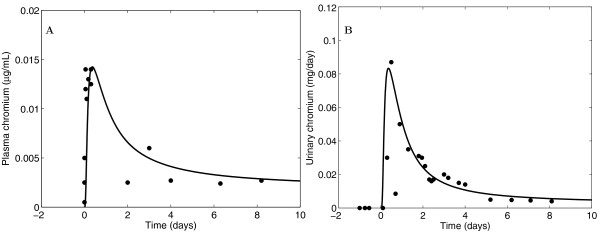
**Comparisons of GTMM predictions with measured human data from the volunteer study by Kerger et al. (1996) **[53]**in which an individual male ingested a 5 mg oral dose of Cr^VI^**. Results are shown for (A) Cr^III ^plasma concentration and (B) Cr^III ^urinary elimination.

Hexavalent chromium (Cr^VI^) is toxic and can lead to a variety of health effects in humans, while trivalent chromium (Cr^III^) is widely considered to be an essential nutrient. Chromium has been detected at numerous hazardous waste sites in the presence of other metals (i.e. in a mixture); individuals living near these sites can be exposed through multiple pathways [50]. Potential synergistic interaction for oxidative stress between chromate and arsenite (leading to DNA damage) has been observed *in vitro *[51]. The wood preservative chromated copper arsenate (CCA) contains a mixture of Cr^VI^, As^V^, and copper, and may pose a health risk to humans [52]. Figure 6 presents a comparison of GTTM predictions with observed data from Kerger et al. (1996) [53], in which a male volunteer orally ingested 5 mg of Cr^VI^. The GTMM incorporated the same parameters as the chromium-specific model by O'Flaherty (2001) [54], which is based on the lead model by the same author (see Additional file 6). Since Cr^VI ^is rapidly reduced to Cr^III ^in the blood, Cr^VI ^is not detectable after a short period of time, hence only Cr^III ^is used for model evaluations.

### Mercury

**Figure 7:**
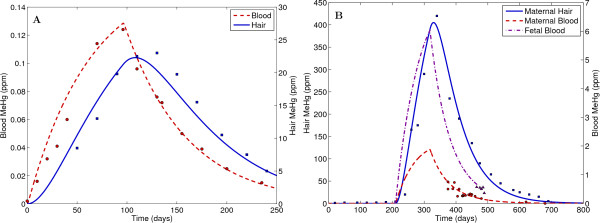
**Comparisons of GTMM predictions with measured human methylmercury (MeHg) data for (A) a male consuming approximately 3 μg/kg/day MeHg for 96 days (Hislop et al., 1983) **[57], **and (B) a pregnant woman consuming 42 μg/kg/day MeHg for 108 days (Amin-Zaki et al., 1976 **[58]). Data legend: hair (blue square), blood (red circle), fetal blood (purple triangle).

Methylmercury enters the food chain from both natural and man-made sources, and high levels are found in ocean and freshwater fish consumed by humans [55]. Methylmercury is a neurotoxin that can pass through the blood brain barrier and the placental barrier. Blood methylmercury levels in infants may be higher than the maternal blood, due to the toxicokinetics of MeHg transport across the placenta. Hence, the PBTK model for methylmercury by Clewell et al. (1999) [56] was focused on women, and included a dynamic fetal subsystem for pregnancy (see Additional file 7). Methylmercury may be excreted in the urine, hair, feces, and breast milk (which becomes a pathway for neonatal exposure), and is also converted to inorganic mercury throughout the body. Relative to other toxic metals, absorption of methylmercury is high and not strongly influenced by essential element status. The GTMM was evaluated using human data from Hislop et al. (1983) [57], for an adult male consuming approximately 3 μg/kg/day MeHg for 96 days. Evaluations were also performed for a pregnant woman and fetus, using data from Amin-Zaki et al. (1976) [58]. The simulation for this case assumed an oral intake of 42 μg/kg/day MeHg, beginning shortly after pregnancy and continuing for 108 days. Simulations for both the male and the pregnant female employed the same physiological and exposure assumptions as the available methylmercury-specific evaluations [59]. Figure 7 presents comparisons of GTMM predictions with the observed data.

### Application of the GTMM to a mixture of metals and non-metals

**Figure 8:**
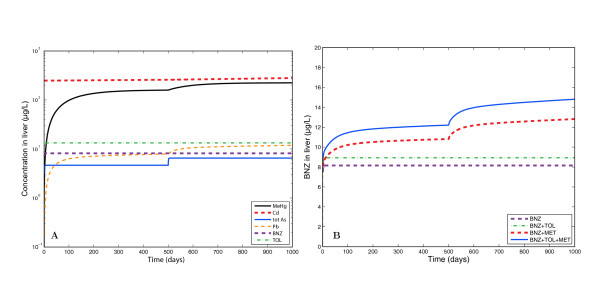
**Hypothetical inhibition of benzene (BNZ) metabolism in the liver by cadmium (Cd), lead (Pb), methylmercury (MeHg), total arsenic (tot As), and toluene (TOL)**. Metal intakes were increased by 40% of the original intakes at day 500. A: Metal and VOC liver concentrations for the base-case (no interactions). B: Benzene liver concentrations under different interaction assumptions.

In order to evaluate the flexibility of the GTMM, it was applied to a hypothetical case-study in which co-exposures to multiple metals and nonmetals were simulated simultaneously by taking into account potential metabolic interactions. Since toxic metal exposures could disrupt the metabolism of a variety of drugs and chemicals [9], the scenario considered involved exposure to a mixture of methylmercury, cadmium, lead, arsenic (and metabolites), toluene, and benzene (Figure 8). The simulation incorporated the potential effect of toxic metals present in the liver on benzene and toluene metabolism, in addition to known competitive inhibition between benzene and toluene [60]. As more metals accumulate in the liver, the rates of metabolism of nonmetals decrease, causing higher accumulation of benzene and toluene. As benzene and toluene concentrations increase, the competitive inhibition between these two chemicals further reduces the rate of metabolism, hence resulting in higher levels of both chemicals. To model a possible effect of toxic metals on the metabolic rate of benzene and toluene, a linear tissue exposure-response model with a short time-lag was used to relate liver metal concentration to a fractional decrease in maximum reaction velocity. For the study purposes, the contributions of each metal to the toxic effect were set to arbitrary values since the actual magnitudes of these interactions are not known. Model parameters were adjusted to give metals with low liver concentrations higher weights in order for each metal to have an approximately equal toxic effect.

The hypothetical case study focuses on a 30-year old male experiencing continuous dietary exposure to metals (15 μg/day cadmium, 40 μg/day methylmercury, 70 μg/day lead, and 100 μg/day inorganic arsenic), and inhalation exposure to volatile organics (20 ppm toluene and 10 ppm benzene). Exposures continued for 500 days, reflecting an approximate steady state. However since the half-life of cadmium in the liver is extremely long, its corresponding steady state levels were estimated using a PBTK model run for an individual from birth to age 30, assuming a cadmium intake of 0.2 μg/kg/day (which is equivalent to 15 μg/day at age 30). The levels of cadmium in all tissues at age 30 were then used as the initial condition for the short-term simulations.

After 500 days, all metal intakes were increased by 40% of their baseline values in order to observe the dynamic (state transition) effects of a variable exposure. Exposure to toluene and benzene remained constant, and was not increased at day 500. Figure 8 (a) shows predicted liver concentrations of cadmium, lead, total arsenic, methylmercury, benzene, and toluene for the base-case (i.e. considering no interactions). Figure 8 (b) shows predicted liver benzene concentration for the base-case scenario and for different interaction assumptions. The increase in benzene concentration beyond day 500 is attributable to increased metal exposure. These results show that, depending on the types of metabolic interactions, there is the potential for substantial increases in the steady-state level of benzene in the liver. It must be noted that the precise relationships between toxic metal exposure and metabolic reaction rates of non-metals is not known and further study is needed in this area.

## Discussion and Conclusions

The previous sections outlined the need, development, implementation, and evaluation of a Generalized Toxicokinetic Modeling system for Mixtures (GTTM), applicable to both metals and non-metals. At the evaluation stage, the implementations of the GTTM for individual chemicals (metals or metal compounds) employed assumptions that were used in the formulations or applications of literature models, but were harmonized via consistent whole body physiology. The GTMM is a step in the on-going development of an integrative toxicokinetic/toxicodynamic system that simulates binary and higher order metal interactions.

The GTMM provides a central component of a novel framework that aims to account for total exposures (cumulative and aggregate) of individuals and populations to mixtures of chemicals; these mixtures can arise from many sources and routes, including environmental releases, use of consumer products, and dietary intake. Specifically, the GTMM has been developed as a component of two complementary and evolving systems that provide the above-mentioned framework: the Modeling ENvironment for TOtal Risk studies (MENTOR) that addresses the "source-to-dose" steps in the exposure and risk modeling sequence [61], and the DOse Response Information ANalysis system (DORIAN) that addresses the biological "dose-to-effect" steps [4]. In the case of MENTOR, the GTMM links to various multimedia/multipathway exposure modules for chemical mixtures, while in the case of DORIAN the GTMM has been designed to provide links to biologically-based dose-response (BBDR) modules for toxicodynamic processes, as these become available.

In addition to providing linkages of PBTK models for metal mixtures with biologically-based dose-response (BBDR) models for toxic effects, the framework should eventually also provide links with PBTK/BBDR models for essential elements. A manganese PBTK model for humans (which is in the early stages of development [62]) can be used to study interactions of toxic and essential metals via the GTMM. For mixtures of metals such as lead, cadmium, and arsenic, there is a need for BBDR models of renal and hepatic effects, because renal dysfunction impacts the elimination of essential and toxic metals in the plasma, and hepatic dysfunction may lead to potential interactions with organics, drugs, PCBs and pesticides. The magnitudes of these interactions *in vivo *are not currently known. However the GTMM can be used to study hypotheses regarding impacts of exposures from multiple metals and nonmetals, and to help identify priority areas for studying environmental health risks from exposures to complex chemical mixtures. The incorporation of whole-body physiology via linkages to up-to-date parameter databases is also useful in examining the distributions of risks within both the general population and selected susceptible subpopulations.

## Competing interests

The authors declare that they have no competing interests.

## Authors' contributions

AFS developed and implemented the GTMM as part of his doctoral research under the joint direction of PGG and SSI. All authors read and approved the final manuscript.

## Supplementary Material

Additional file 1 **Table of parameter values for cadmium**. Model constants and parameter descriptions for the cadmium toxicokinetic model. Click here for file

Additional file 2 **Figure of cadmium toxicokinetic model**. Model schematic including kinetic constants for the cadmium toxicokinetic model. Click here for file

Additional file 3 **Table of partition coefficients for arsenic**. Model constants (partition coefficients) for the arsenic toxicokinetic model. Click here for file

Additional file 4 **Table of metabolic constants for arsenic**. Model constants (absorption, metabolism, and elimination) and descriptions for the arsenic toxicokinetic model. Click here for file

Additional file 5 **Table of parameter values for lead**. Model constants and parameter descriptions for the lead toxicokinetic model. Click here for file

Additional file 6 **Table of parameter values for chromium**. Model constants and parameter descriptions for the chromium toxicokinetic model. Click here for file

Additional file 7 **Table of parameter values for methylmercury**. Model constants and parameter descriptions for the methylmercury toxicokinetic model. Click here for file
