# Supplementary material for: A generalized physiologically-based toxicokinetic modeling system for chemical mixtures containing metals
Source: Theor Biol Med Model. 2010 Jun 2;7:17. doi: 10.1186/1742-4682-7-17 (PMC2903511; doi:10.1186/1742-4682-7-17)
Supplement: Additional file 1 — Table of parameter values for cadmium. Model constants and parameter descriptions for the cadmium toxicokinetic model. [file 1742-4682-7-17-S1.PDF]

PBTK model parameters for cadmium, adapted from Choudhury et al. (2001)[1]

Constant	Value	Definition
k1	0.1 (cigarette), 0.7 (dust)	Fraction swallowed after deposition and clearance from nasopharyngeal/tracheobronchial compartments
k2	0.4 (cigarette), 0.13 (dust)	Fraction of inhaled dust/smoke deposited in alveolar compartment
k3	0.05/day	Rate constant for absorption by system from lung
k4	$0.1\times$ k3	Rate constant for clearance to the G.I. tract from respiratory system
k5	0.05 (men), 0.10 (women)	Fraction absorbed to GI tract and systemic circulation
k6	0.05/day	Rate constant for absorption to system from G.I.
k7	0.25	Fraction of cadmium absorbed by system that is up taken to blood-3 compartment
k8	1 μg/day	Maximum rate that can be up taken to blood-3 compartment
k9	0.44	Fraction transferred from blood-1 to other tissues
k10	$1.4\text{x}10^{-4}$/day	Rate constant for transfer from other tissues to blood-1
k11	0.27	Fraction transferred from blood-1 to feces
k12	0.25	Fraction transferred from blood-1 to liver
kx*	0.04	Fraction transferred from blood-1 to blood-2
k13	$3.0\text{x}10^{-5}$/day	Rate constant for transfer from liver to blood-1
k14	$1.6\text{x}10^{-4}$/day	Rate constant for transfer from liver to blood-3
k15	$5.0\text{x}10^{-5}$/day	Rate constant for transfer from liver to feces
k16†	0.012/day	Rate constant for transfer from blood-2 to blood-3
k17‡	0.95	Fraction transferred from blood-3 to kidneys
k18	$1\text{x}10^{-5}$/day	Rate constant for transfer from kidney to blood-1
k19⊕	$1.4\text{x}10^{-4}$/day	Rate constant for transfer of cadmium from kidney tubules to urine
k21	$1.1\text{x}10^{-6}$/day	Factor for increased transfer to urine after age 30

* kx = (1-k9-k11-k12)

† Red blood cell decay (83 day half-life)

‡ Decreases from age 30 to 80 by 33%. Fraction transferred from blood-3 to urine is (1-k17)

⊕ Increases from age 30 by k21

[1] Choudhury H, Harvey T, Thayer WC, Lockwood TF, Stiteler WM, Goodrum PE, Hassett JM, Diamond GL: **Urinary cadmium elimination as a biomarker of exposure for evaluating a cadmium dietary exposure–biokinetics model**. *Journal of Toxicology and Environmental Health-Part A* 2001, **63**(5):321–50.
