# Supplementary material for: A generalized physiologically-based toxicokinetic modeling system for chemical mixtures containing metals
Source: Theor Biol Med Model. 2010 Jun 2;7:17. doi: 10.1186/1742-4682-7-17 (PMC2903511; doi:10.1186/1742-4682-7-17)
Supplement: Additional file 2 — Figure of cadmium toxicokinetic model. Model schematic including kinetic constants for the cadmium toxicokinetic model. [file 1742-4682-7-17-S2.PDF]

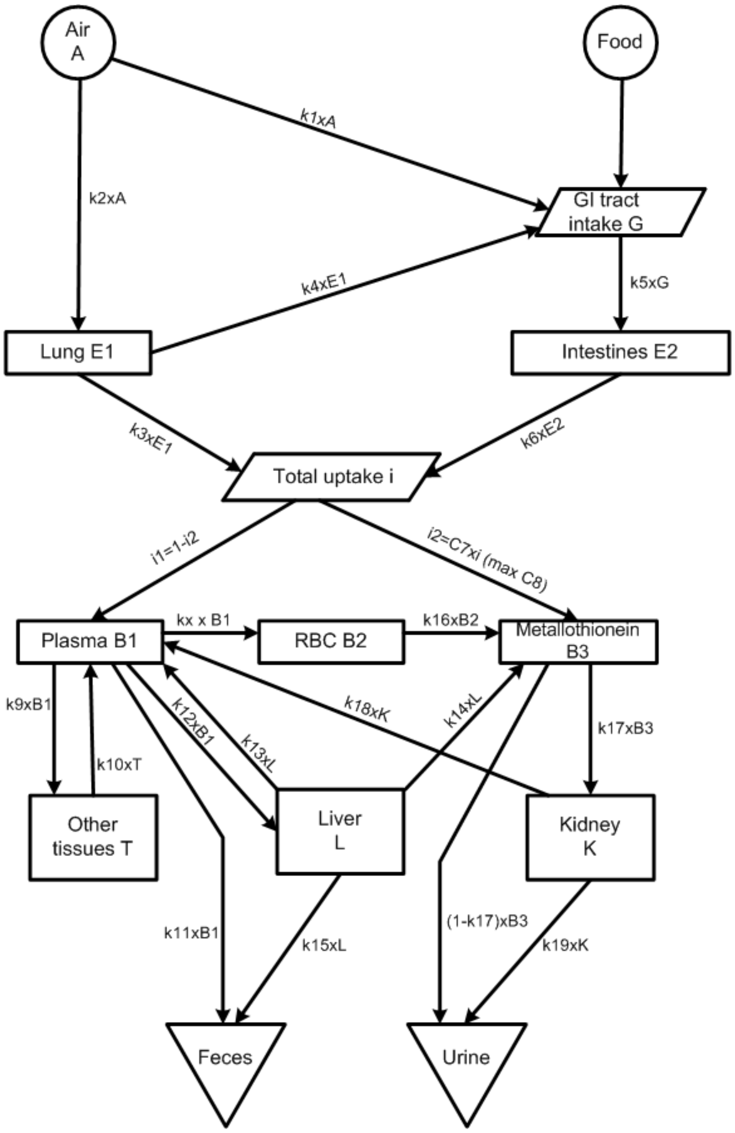

Figure 1: Toxicokinetic model structure for cadmium (Cd).[1] Blood perfusion is neglected, and transport occurs via albumen (blood-1), erythrocytes (blood-2), and metallothionein binding (blood-3).

1. Nordberg GF, Kjellstrom T: **Metabolic model for cadmium in man**. *Environ Health Perspect* 1979, **28**:211–7.
