# Supplementary material for: A generalized physiologically-based toxicokinetic modeling system for chemical mixtures containing metals
Source: Theor Biol Med Model. 2010 Jun 2;7:17. doi: 10.1186/1742-4682-7-17 (PMC2903511; doi:10.1186/1742-4682-7-17)
Supplement: Additional file 3 — Table of partition coefficients for arsenic. Model constants (partition coefficients) for the arsenic toxicokinetic model. [file 1742-4682-7-17-S3.PDF]

Tissue/blood partition coefficients for arsenic species for the El-Masri/Kenyon model[1]

Tissue	As^{V}	As^{III}	MMA^{V}	DMA^{V}
Small Intestine	2.7	8.3	2.2	2.1
Skin	7.9	7.4	2.61	2.4
Brain	2.4	2.4	2.2	3.3
Muscle	7.9	7.4	2.61	2.4
Kidney	8.3	11.7	4.4	3.8
Liver	15.8	16.5	3.3	3.3
Lung	2.1	6.7	1.3	1.3
Heart Muscle	7.9	7.4	2.61	2.4

1. El-Masri HA, Kenyon EM: **Development of a human physiologically based pharmacokinetic (PBPK) model for inorganic arsenic and its mono- and di-methylated metabolites**. *J Pharmacokinet Pharmacodyn* 2007.
