# Supplementary material for: A generalized physiologically-based toxicokinetic modeling system for chemical mixtures containing metals
Source: Theor Biol Med Model. 2010 Jun 2;7:17. doi: 10.1186/1742-4682-7-17 (PMC2903511; doi:10.1186/1742-4682-7-17)
Supplement: Additional file 4 — Table of metabolic constants for arsenic. Model constants (absorption, metabolism, and elimination) and descriptions for the arsenic toxicokinetic model. [file 1742-4682-7-17-S4.PDF]

Kinetic parameters for the El-Masri/Kenyon arsenic PBTK model[1]

| Parameter | Value | Units |
|---|---|---|
| Oral absorption | | |
| $As^{V}$ | 0.003 | $min^{-1}$ |
| $As^{III}$ | 0.004 | $min^{-1}$ |
| $MMA^{V}$ | 0.007 | $min^{-1}$ |
| $DMA^{V}$ | 0.007 | $min^{-1}$ |
| Urinary excretion | | |
| $As^{V}$ and $As^{III}$ | 0.07 | $min^{-1}$ |
| $MMA^{V}$ and $MMA^{III}$ | 0.3 | $min^{-1}$ |
| $DMA^{V}$ and $DMA^{III}$ | 0.13 | $min^{-1}$ |
| Reduction | | |
| $As^{V} \rightarrow As^{III}$ | 0.003 | $min^{-1}$ |
| $MMA^{V} \rightarrow MMA^{III}$ | 0.008 | $min^{-1}$ |
| $DMA^{V} \rightarrow DMA^{III}$ | 0.004 | $min^{-1}$ |
| Oxidation | | |
| $As^{III} \rightarrow As^{V}$ | 0.25 | unitless |
| $MMA^{III} \rightarrow MMA^{V}$ | 0.63 | unitless |
| $DMA^{III} \rightarrow DMA^{V}$ | 0.65 | unitless |
| Methylation | | |
| Vmax ($As^{III} \rightarrow MMA^{V}$) | $5.3\text{x}10^{-7}$ | mole/min |
| Km ($As^{III} \rightarrow MMA^{V}$) | $3\text{x}10^{-6}$ | mole/L |
| Vmax ($As^{III} \rightarrow DMA^{V}$) | $2\text{x}10^{-6}$ | mole/min |
| Km ($As^{III} \rightarrow DMA^{V}$) | $3\text{x}10^{-6}$ | mole/L |
| Vmax ($MMA^{III} \rightarrow DMA^{V}$) | $6.6\text{x}10^{-7}$ | mole/min |
| Km ($MMA^{III} \rightarrow DMA^{V}$) | $3\text{x}10^{-6}$ | mole/L |
| Ki* | $4\text{x}10^{-5}$ | mole/L |

*Noncompetitive inhibition constant for $MMA^{III}$ inhibiting $As^{III}$ methylation, and $As^{III}$ inhibiting $MMA^{III}$ methylation


1. El-Masri HA, Kenyon EM: **Development of a human physiologically based pharmacokinetic (PBPK) model for inorganic arsenic and its mono- and di-methylated metabolites**. *J Pharmacokinet Pharmacodyn* 2007.
