# Supplementary material for: A generalized physiologically-based toxicokinetic modeling system for chemical mixtures containing metals
Source: Theor Biol Med Model. 2010 Jun 2;7:17. doi: 10.1186/1742-4682-7-17 (PMC2903511; doi:10.1186/1742-4682-7-17)
Supplement: Additional file 5 — Table of parameter values for lead. Model constants and parameter descriptions for the lead toxicokinetic model. [file 1742-4682-7-17-S5.PDF]

PBTK model parameters for lead in humans, adapted from O'Flaherty (1995)[1]

Parameter	Value	Notes
Partition coefficients		
Liver/plasma	50	
Kidney/plasma	50	
Rapidly perfused/plasma	50	
Poorly perfused/plasma	2	
Kinetic parameters		
Plasma/bone clearance	15000	Fractional clearance of lead from plasma into forming bone (L plasma cleared/L bone formed)
RBC bind capacity	2.7	Maximum capacity of sites in red blood cells to bind lead (mg/L of RBC volume)
RBC bind saturation	0.0075	Half-saturation concentration of lead for binding by sites in red blood cells (mg/L of RBC volume)
RBC unbound linear	1.2	Linear parameter for unbound lead in red blood cells
Fractional GI absorption	variable	Function of age and gender
Fractional lung absorption	0.5	
Urinary clearance	variable	Function of glomerular filtration rate
Bone parameters		
D Diffusion constant	$5e^{-7}$	Diffusion within bone (cm/day/$0.5e^{-4}$ cm)
R Permeability constant	$5e^{-7}$	Diffusion from bone to canalicule (cm/day/$0.5e^{-4}$ cm)
P Permeability constant	0.02	Diffusion from canalicule to bone (cm/day/$0.5e^{-4}$ cm)

This table contains only a partial list of parameters. For a full description of this model, the reader is directed to O'Flaherty (2000),[2] which outlines the model equations and variables in the Advanced Continuous Simulation Language (ACSL).

1. O'Flaherty EJ: **Physiologically based models for bone-seeking elements. V. Lead absorption and disposition in childhood**. *Toxicol Appl Pharmacol* 1995, **131**(2):297–308.

2. O'Flaherty EJ: **Modeling normal aging bone loss, with consideration of bone loss in osteoporosis**. *Toxicol Sci* 2000, **55**:171–88.
