# Supplementary material for: A generalized physiologically-based toxicokinetic modeling system for chemical mixtures containing metals
Source: Theor Biol Med Model. 2010 Jun 2;7:17. doi: 10.1186/1742-4682-7-17 (PMC2903511; doi:10.1186/1742-4682-7-17)
Supplement: Additional file 6 — Table of parameter values for chromium. Model constants and parameter descriptions for the chromium toxicokinetic model. [file 1742-4682-7-17-S6.PDF]

PBTK model parameters for chromium in humans, based on the model by O'Flaherty (2001)[1]

Parameter	Value	Notes
Diffusion		
KRBC3	12	Clearance of diffusible Cr^{III} between plasma/RBC (L/day)
Kd3	3	Diffusion clearance between plasma and all tissues for Cr^{III}
Kd6	30	Diffusion clearance between plasma and all tissues for Cr^{VI}
CR3	5	Fractional deposition of Cr^{III} into forming bone
CR6	15	Fractional deposition of Cr^{VI} into forming bone
Kinetics		
KREDKL	500	Reduction of Cr^{VI} to Cr^{III} in liver and kidney (day^{-1})
KREDBP	0.2	Reduction of Cr^{VI} to Cr^{III} in plasma (day^{-1})
KREDRC	7	Reduction of Cr^{VI} to Cr^{III} in RBCs (day^{-1})
KREDGI	100	Reduction of Cr^{VI} to Cr^{III} in GI tract (day^{-1})
KREDO	5	Reduction of Cr^{VI} to Cr^{III} in all other tissues (day^{-1})
KGI3	0.25	Absorption of Cr^{III} in GI tract (day^{-1})
KGI6	2.5	Absorption of Cr^{VI} in GI tract (day^{-1})
KFX	14	Transport of Cr from GI tract (day^{-1})
KLOSS3B	0.023	Turnover of Cr^{III} from bound diffusible RBC form (day^{-1})
cl	12	Clearance for saturable urinary excretion (L/day)
vm	0.18/0.008†	Vmax for decrease in urinary excretion rate (mg/day)
km	0.015/0.0008†	Km for decrease in urinary excretion rate (mg/L)

†Two sets of parameters were required to fit data from different individuals

This table contains only a partial list of parameters. For a full description of this model, the reader is directed to O'Flaherty (2001),[1] which outlines the model equations and variables in the Advanced Continuous Simulation Language (ACSL).

1. O'Flaherty EJ, Kerger BD, Hays SM, Paustenbach DJ: **A physiologically based model for the ingestion of chromium(III) and chromium(VI) by humans**. *Toxicol Sci* 2001, **60**(2):196–213.
