# Supplementary material for: A generalized physiologically-based toxicokinetic modeling system for chemical mixtures containing metals
Source: Theor Biol Med Model. 2010 Jun 2;7:17. doi: 10.1186/1742-4682-7-17 (PMC2903511; doi:10.1186/1742-4682-7-17)
Supplement: Additional file 7 — Table of parameter values for methylmercury. Model constants and parameter descriptions for the methylmercury toxicokinetic model. [file 1742-4682-7-17-S7.PDF]

PBTK model parameters for methylmercury, adapted from Shipp et. al. (2000)[1]

Parameter*	Mean	CV	Notes
Partition coefficients			
Brain/blood	3.0	0.30	
Brain blood/plasma	1.0	0.30	
Fat/blood	0.15	0.30	
Fetal plasma/placenta	2.0	0.30	
Gut/blood	1.0	0.70	
Hair/blood	248.66	0.70	
Kidney/blood	4.0	0.30	
Liver/blood	5.0	0.30	
Placenta/blood	2.0	0.30	
RBC/plasma	12.0	0.30	
Fetal RBC/plasma	14.0	0.30	
Rapidly perfused/blood	1.0	0.30	
Slowly perfused/blood	2.0	0.30	
Kinetic parameters			
iHg to brain	5.0×10^{-5}	0	iHg from kidneys to brain, L/h/$kg^{0.75}$
Loss of iHg from brain	0.001	0	iHg from brain to kidneys, L/h/$kg^{0.75}$
Brain MeHg to iHg	1.2×10^{-5}	0.30	De-methylation of MeHg, h^{-1}
Biliary clearance of MeHg	0.0001	0.30	MeHg from liver to intestine, L/h/$kg^{0.75}$
Brain/brain plasma MeHg diffusion	0.01	0.30	Blood-brain barrier transport, L/h/$kg^{0.75}$
Intestinal MeHg to iHg	0.0001	0.30	De-methylation of MeHg, L/h/$kg^{0.75}$
Fecal excretion of MeHg	0.0002	0.36	From intestine, L/h/$kg^{0.75}$
Excretion of MeHg into hair	7.0×10^{-6}	0.25	L/h/$kg^{0.75}$
Liver MeHg to iHg	1.0×10^{-5}	0.30	L/h/$kg^{0.75}$
RBC/plasma MeHg diffusion	1.5	0.30	L/h/$kg^{0.75}$
Intestinal reabsorption of MeHg	0.005	0.30	From intestine to gut, L/h/$kg^{0.75}$
Fetal kinetic parameters			
Placenta/fetal MeHg diffusion	1.0	0.50	L/h
Fetal RBC/plasma diffusion	100.0	0.50	L/h/$kg^{0.75}$

*All distributions presented in this table are lognormal

1. Shipp AM, Gentry PR, Lawrence G, Van Landingham C, Covington T, Clewell HJ, Gribben K, Crump K: **Determination of a site-specific reference dose for methylmercury for fish-eating populations**. *Toxicol Ind Health* 2000, **16**(9-10):335–438.
